# Quantum mechanical modeling of high-intensity laser pulse interaction with hydrogen atom with considering the magnetic field and polarization

**DOI:** 10.1038/s41598-024-59515-4

**Published:** 2024-04-18

**Authors:** Marjan Zakavi, Mohammad Sabaeian

**Affiliations:** 1 Department of Physics, Faculty of Science, Shahid Chamran University of Ahvaz, Ahvaz, Iran; 2 Center for Research on Laser and Plasma, Shahid Chamran University of Ahvaz, Ahvaz, Iran

**Keywords:** Attosecond science, Theoretical physics

## Abstract

In the study of the non-relativistic interaction between high-intensity femtosecond laser pulses and atoms, the influence of the magnetic field is commonly overlooked. This work investigates the effects of the magnetic field in the high-intensity few-cycle laser pulses with non-relativistic intensity of $3.5 \times 10^{14} { }\;\;{\text{W}}/{\text{cm}}^{2}$ at the center wavelength of 800 nm on the high-order harmonic generation (HHG), attosecond pulse train (APT), isolated attosecond pulse (IAP), and the electron trajectory in the hydrogen atom, employing the numerical solution of the time-dependent Schrödinger equation in three dimensions (3D-TDSE). Two polarizations, linear and circular, are considered. A comparison with the scenario where the magnetic field is not considered shows that the magnetic field can apply significant corrections to the results. Particularly, considering the magnetic field for circular polarization can make the cutoff frequency of HHG coincide with the semi-classical relationship of $\hbar {\upomega }_{{\text{c}}} = {\text{I}}_{{\text{p}}} + 3.17{\text{U}}_{{\text{p}}}$, a case that for circular polarization does not exist without considering the magnetic field. Moreover, accounting for the magnetic field leads to a reduction in the attosecond pulse duration for circular polarization for APT ($360{\text{ as}}$ versus $241{\text{ as}}$) and for IAP ($834{\text{ as}}$ versus $602{\text{ as}}$). Additionally, the difference in production efficiency of HHG and APT between linear and circular polarization is reduced by two orders of magnitude, when magnetic field is considered. Although considering the magnetic field complicates the electron trajectory, especially for circular polarization, however, our quantum model provides enhanced insight into how the interaction works, especially when and where the electron collides with the parent nucleus. In this case, the quantum mechanical modeling largely covers the huge difference of not considering the magnetic field in the results predicted by other works.

## Introduction

High-order harmonic generation (HHG) is an extreme nonlinear process arising from the interaction of strong field laser pulses with atoms and molecules in the gaseous phase. When the correct phase matching is provided, HHG leads to the production of coherent attosecond pulse trains (APT) and/or isolated attosecond pulse (IAP) in the XUV to soft X-ray region. These short pulses enable the study of electron dynamics on its inherent time scale^1,2^. HHG has many other applications, including imaging of molecular orbitals^3,4^, attosecond spectroscopy^5,6^, attochemistry^7–9^, etc.

The HHG spectrum consists of four parts: (1) the fundamental part, which corresponds to the frequency of the driving laser with high intensity; (2) the perturbative part, containing a few harmonic orders; (3) the non-perturbative part, consisting of high-order harmonics with approximately equal intensity, known as the plateau; and (4) the cut-off region, where there is a rapid decline at the end of the plateau. The plateau region in the HHG spectrum can be utilized to generate an APT or IAP. To generate an APT, one can apply a Fourier transform (FT), if a suitable phase relation among the harmonics is provided, and other regions outside the plateau are filtered out. For IAP, some methods such as polarization gating or attosecond lighthouse are used. We, in this work, apply FT on the cutoff region, in which by keeping the cutoff region and filtering out other regions, the IAP is generated^10^.

HHG in the gaseous medium can be well described by a three-step model^11,12^. This three-step model provides a conceptual framework for understanding HHG, which is a fascinating process wherein intense laser pulses interact with atoms, resulting in the generation of high-frequency light. In the three-step model, the process begins with an electron being ionized from an atom by the laser pulse. The electron is then accelerated by the laser field and reaches high energy states. Finally, the electron is driven back toward the atom, and upon recombination, it releases its excess energy in the form of a high-energy photon, which is a harmonic of the laser frequency. This model has proven to be a useful tool for understanding the physics underlying HHG and has led to many insightful experiments and theoretical analyses. The maximum energy emitted when an electron recombines with the nucleus (cutoff frequency) is given by $\hbar {\upomega }_{{\text{c}}} = {\text{I}}_{{\text{p}}} + 3.17{\text{U}}_{{\text{p}}}$^13^, where ${\text{I}}_{{\text{p}}}$ is the ionization potential and ${\text{U}}_{{\text{p}}}$ is the ponderomotive energy. Ponderomotive energy is the energy associated with the oscillation or motion of a charged particle in an electromagnetic field. It is a form of kinetic energy that arises due to the interaction between the field and the charged particle, which is given as ${\text{U}}_{{\text{p}}} = \frac{{{\text{e}}^{2} {\text{E}}_{0}^{2} }}{{4{\text{m}}_{{\text{e}}} {\upomega }^{2} }}$, where ${\text{E}}_{0}$ is the electric field amplitude of the driving laser field and ${\upomega }$ is its frequency. While ${\text{m}}_{{\text{e}}}$ and ${\text{e}}$ are the mass and charge of the electron, respectively^14,15^.

The study of the interaction of strong field laser pulses with atoms can be divided into perturbative and non-perturbative regimes^16^. For intensities lower than $1 \times 10^{13} \;\;{\text{W}}/{\text{cm}}^{2}$, the perturbative description is sufficient, whereas, for intensities above $1 \times 10^{14} \;\;{\text{W}}/{\text{cm}}^{2}$, where the potential resulting from the driving laser field exerting on the atom becomes comparable to the Coulomb potential of the atom, the non-perturbation description is mandatory^17,18^.

The study of non-perturbative interactions is mainly based on two models: (1) the quantum mechanical model and (2) the classical model. The time-dependent Schrodinger equation (TDSE) is used to study the interaction of laser pulses with atoms, as it accurately captures the time evolution of the system’s wave function. When a laser pulse interacts with an atom, it can induce transitions between different energy levels of the atom or even ionize the atom^18^. These processes occur in the time domain and can be accurately described by TDSE which considers the time-dependent Hamiltonian of the system, including the interaction between the laser pulse and the atom. As a result, it provides a complete picture of the dynamics of the system, including the amplitude and phase of the wave function at each point in time. This information can be used to calculate various physical quantities of interest, such as the ionization probability or the energy transferred from the laser pulse to the atom. In the quantum model, to directly solve the TDSE without using approximations, numerical solutions are preferred. In this approach, the system is considered as a non-interacting ensemble of atoms or molecules. There are several approaches to solving the TDSE; Rong-Kutta^19^, Crank–Nicholson^20–24^, split operator^25–30^, split operator and Rung-Kutta^31^, are some strong approaches. On the other hand, the classical model uses Maxwell’s equations. The particle-in-cell (PIC) method is based on Maxwell’s equations and is used to simulate the collective behavior of atoms^32–36^.

Here, we focus on studying the interaction of a hydrogen atom with non-relativistic few-cycle femtosecond laser pulses in three dimensions. Although many articles have been published in this field, precise solutions for this issue still present challenges. As stated in Ref.^37^, “the solution of 3D-TDSE to find the time evolution of wave function even for a single electron atom is still a formidable computational challenge”. The solution of the 3D-TDSE for hydrogen atoms has been reported by Patchkovskii et al.^37^, and in the strong field approximation (SFA) by Xie et al.^38^, Birulia et al.^39^, Murakami et al.^40^, and Neyra et al.^41^.

To do as stated, we avoid approximations as much as possible. In particular, we include the magnetic field in the calculations. Naturally, considering the magnetic field makes it impossible to solve the problem analytically. Therefore, we use the numerical method to solve the Schrödinger equation. Also, we consider two types of polarization, linear and circular, for the interacting field. By solving the 3D-TDSE, we investigate the time evolution of electron probability density, dipole acceleration, spectrum of HHG, ATP, IAP, and the trajectory of electron under the pulse intensity of $3.5 \times 10^{14} { }\;\;{\text{W}}/{\text{cm}}^{2}$. As we discuss below, the method we use to solve numerically the 3D-TDSE is quite simple. The key point in this method is the accurate selection of the numerical method and the meshing of the temporal and spatial dimensions to solve the equation using the finite difference method. Similarly, the selection of boundary conditions and the position of their application on the wave function is also important. Our investigations show that if the mentioned parameters are not selected accurately, the answer will be divergent or inaccurate. Our criteria to ensure the accuracy of the answers are the experimental results and theoretical works of other researchers.

We show that the production efficiency of HHG is higher for linear polarization compared to circular polarization, as mentioned also in Refs.^42–44^. Nevertheless, by considering the magnetic field, the difference in efficiency decreases, but still, the values for linear polarization are higher than circular polarization. Some studies have considered the role of the magnetic field in the intensity ranges of $> 1 \times 10^{16} \;\;{\text{W}}/{\text{cm}}^{2}$ for ${{ \lambda }} = 800{\text{ nm}}$^45,46^, which are in the relativistic intensity range. An interesting point to note here is the mismatch of the cutoff frequency for circular polarization with that predicted by classical model, when the magnetic field is not taken into account. By considering the magnetic field, the cutoff frequency would be in good agreement with the cutoff low. It is common that in the intensities lower than $1 \times 10^{16} \;\;{\text{W}}/{\text{cm}}^{2}$ and for ${\uplambda } = 800{\text{ nm}}$, the magnetic field is completely ignored in calculations^47–49^, apart from the role of magnetic field considered by Kim et al.^50^, for linear polarization. Another interesting result of this study is the path of the electron around the parent nucleus during the interaction with the laser field. Due to the Lorentz force, this path is complex in three dimensions. However, the answers clearly show that the electron hits the nucleus after many twists and turns.

As mentioned, we base our approach on the 3D solution of the TDSE. We use the symmetric Euler method (central deference) up to ${\text{O}}\left( {{\text{h}}^{2} } \right)$ for the numerical solution of 3D-TDSE. To reach accurate solutions, we use boundary conditions of mask function and proper time and space meshes. Choosing appropriate space and time meshes is essential. We try not to enter any approximation into the calculations. All calculations are carried out in Cartesian coordinates and atomic units (${\text{a}}.{\text{u}}.$). To ensure that the wave function does not diverge, the normalization of the wave function was checked throughout the code execution. The codes were written in Intel Fortran and run with the Linux operating system.

## Results and discussion

In this section, we present the results of our numerical calculations for both cases: considering and ignoring the magnetic field in the Schrödinger equation. We examine both linear and circular polarizations for the driving laser field.

### Hydrogen’s wave function, electron trajectory, HHG, APT, and IAP without considering magnetic field

In this section, we focus on the interaction of non-relativistic laser pulse with hydrogen atom, by ignoring the magnetic field effect. The laser intensity used is $3.5 \times 10^{14} \;\;{\text{W}}/{\text{cm}}^{2}$, which is a common value for non-relativistic laser intensity. The wavelength of the laser is set to ${\uplambda } = 800{\text{ nm}}$, which is typical for Ti:sapphire laser output. The laser pulse is considered to be a few-cycle pulse, with a full width at half maximum (${\text{FWHM}}$) of $4.35{\text{ fs}}$. Both linear and circular polarizations are studied in the simulation.

For simulation, the time interval is chosen as $\left[ {0,5{\text{T}}} \right]$, where ${\text{T}} = 2{\uppi }/{\upomega }$, and ${\upomega } = 0.057{\text{ a}}.{\text{u}}.$. This corresponds to a time interval of $13.32{\text{ fs}}$. The time mesh is set to $1/160{\text{ a}}.{\text{u}}.$, and the spatial mesh is set to $1/2{\text{ a}}.{\text{u}}..$ The ${\text{x}}$ and ${\text{y}}$ intervals are the same and are $\left[ { - 75,75} \right]{\text{ a}}.{\text{u}}.$. Since we are ignoring the magnetic field in this section, there is no need to use the ${\text{z}}$-axis in the calculations. To produce a linear polarized field, we set ${\text{C}} = 1$ and ${\text{D}} = 0$ in Eq. (3). For circular polarization, we set ${\text{C}} = {\text{D}} = 1/2$. The CEP value was set to zero ($\phi = 0$).

**Figure 1:**
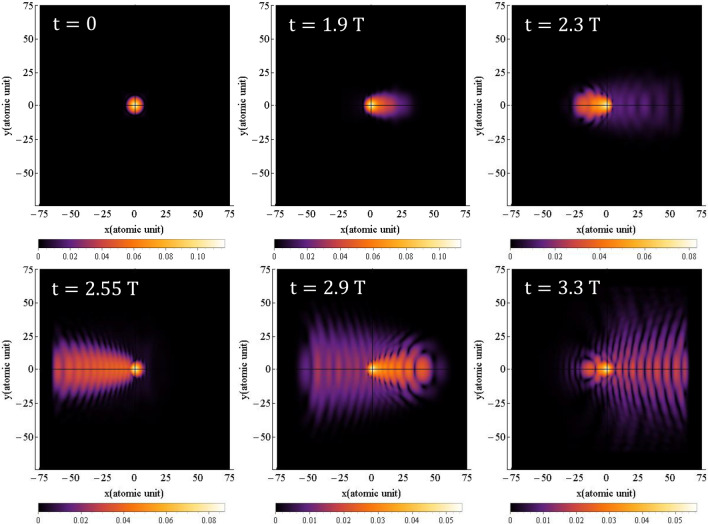
The probability density of the electron around the nucleus under linear polarization, when the magnetic field is not considered for ${{\varvec{\uplambda}}} = 800 \;\;{\mathbf{nm}}$ and the intensity of $3.5 \times 10^{14} \;\;{\mathbf{w}}/{\mathbf{cm}}^{2}$. The direction of the electric field is along the ${\mathbf{x}}$ axis. The ${\mathbf{x}}$ and ${\mathbf{y}}$ intervals are both the same as $\left[ { - 75, 75} \right]$.

**Figure 2:**
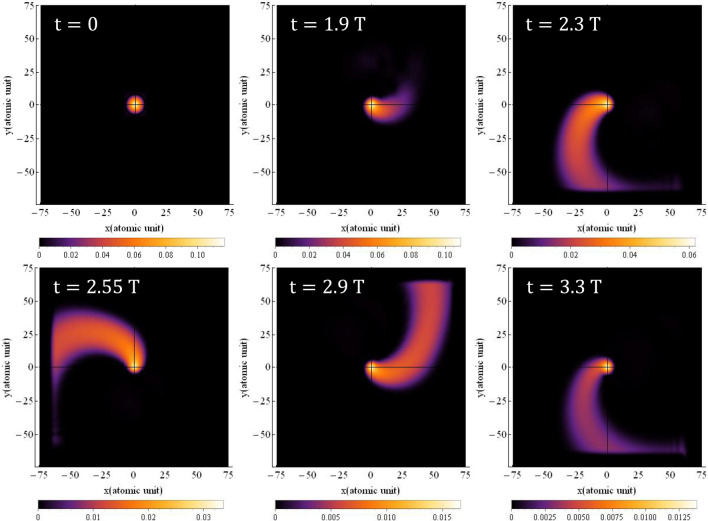
The probability density of the electron around the nucleus for circular polarization, when the magnetic field is not considered for ${{\varvec{\uplambda}}} = 800\;\; {\mathbf{nm}}$ and the intensity of $3.5 \times 10^{14} \;\;{\mathbf{w}}/{\mathbf{cm}}^{2}$. The direction of the electric field is in the ${\mathbf{xy}}$-plane. The ${\mathbf{x}}$ and ${\mathbf{y}}$ intervals are both the same as $\left[ { - 75, 75} \right]$.

The probability density of finding the electron around the nucleus is obtained by squaring the time-dependent hydrogen's wave function, $\left| {{\uppsi }\left( {{\vec{\text{r}}},{\text{t}}} \right)} \right|^{2}$, which is obtained from Eq. (1). The results for simulating the time-dependent probability density of the electron are presented in Figs. 1 and 2, for linear and circular polarization, respectively. The figures show the probability density at six different times of $0,{ }1.9{\text{ T}},{ }2.3{\text{ T}},{ }2.55{\text{ T}},{ }2.9{\text{ T}}$, and $3.3{\text{ T}}$.

In the supplementary material, the probability densities are presented as video files, video_1 and video_2, where the simulation time has been divided into $100$ frames, such that each frame has been captured every $133{\text{ as}}$.

Figure 1 clearly illustrates the oscillations of the electron's probability density around the nucleus. As expected, due to the linear polarization of the field, the axis of oscillation remains unchanged. The oscillation of the electron’s wave function in time leads to an increase in its amplitude in the transverse direction. These oscillations represent the electron transition from lower to higher-energy quantum states. If the electric field has enough time, which depends on the wavelength or frequency of the field, the electron enters continuum states. Subsequently, by altering the direction of the field, the electron undergoes acceleration towards the parent ion and eventually collides with it. Our quantum model comprehensively calculates and presents all these steps, including tunneling, acceleration in the field, and interaction with the parent ion.

The small radius of the hydrogen atom ($0.51{ }$ Å), in contrast to the much larger path of the electron in the field, renders tunneling in the figure barely detectable. The cutoff frequency is directly proportional to the square of electron's time-of-flight in the laser field. With a FWHM of $4.35{\text{ fs}}$, the laser pulse contains approximately one and a half optical cycles. This implies that each pulse can detach the electron three times during each interaction with the atom. However, the spatial interval within which the wave function oscillates needs to be limited to account for computational constraints, such as run time and required RAM. In such cases, appropriate boundary conditions should be applied to optimize the run time and RAM usage. Reflections of wave functions from the boundaries, even for the far boundaries, can interfere with outgoing waves, leading to high constructive peaks that can distort the results. To mitigate this, the use of a mask function in regions far enough from the atom helps dampen reflections and prevents interference. We assess the appropriateness of the chosen boundaries and the application of the mask function based on the alignment of our results with experimental values, which will be discussed later. This probability density is observed by Derbov and Teper^51^, Amini et al.^52^, Fu et al.^53^ and Petrovi´c et al.^54^.

Figure 2 presents the electron's probability density around the nucleus for a circular polarized field which is obtained by solving Eq. (1). As depicted, the electric field induces rotation of the electron's wave function around the nucleus, accompanied by an increase in its transverse expansion. This raises the question of how the electron can collide with the nucleus while its wave function is rotating around it. Naturally, the semi-classical three-step model cannot offer a convincing answer in this regard, unless it suggests that the likelihood of collision with the nucleus is lower compared to the case of linear polarization. Nonetheless, in order to provide a quantitative answer to this question using a quantum mechanical model, we have undertaken calculations to determine the path of the electron's movement around the nucleus.

**Figure 3:**
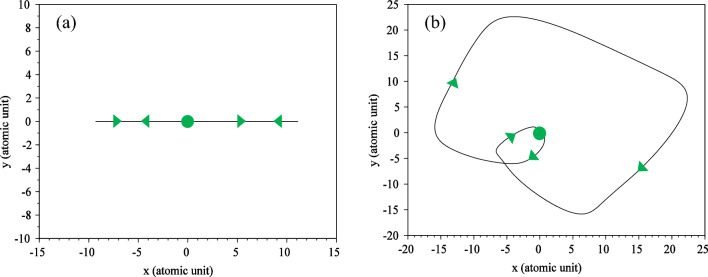
The electron trajectory for (**a**) linear and (**b**) circular polarization. Solid dots indicate the starting point and triangles the direction of electron motion. The electric field direction is along the ${\text{x}}$ axis for linear polarization and in the ${\text{xy}}$-plane for circular polarization. The ${\text{x}}$ and ${\text{y}}$ intervals are both the same as $\left[ { - 75,{ }75} \right]$. The wavelength is $800{\text{ nm}}$ with an intensity of $3.5 \times 10^{14} \;\;{\text{W}}/{\text{cm}}^{2}$ for the laser pulse**.**

The electron trajectory, as depicted in Fig. 3, provides a classical representation of a quantum phenomenon, which is obtained by solving Eq. (8). In Fig. 3a, which corresponds to the linear polarization, the trajectory of the electron oscillates along a straight line. The path of the trajectory closely resembles the motion of a simple oscillator, which aligns well with its classical counterpart. However, for circular polarization, as shown in Fig. 3b, a more intriguing outcome emerges. The electron's path becomes somewhat intricate, forming a series of circles with unequal radii that eventually converge towards the parent nucleus. In a circularly polarized field, the electron follows a spiral trajectory, ultimately colliding with the nucleus. Although our calculations were performed for a pulse spanning one and a half optical cycles, we only observe one collision. It appears that the other peaks of the driving laser pulse were not able to induce significant collisions with the parent nucleus for the electron.

**Figure 4:**
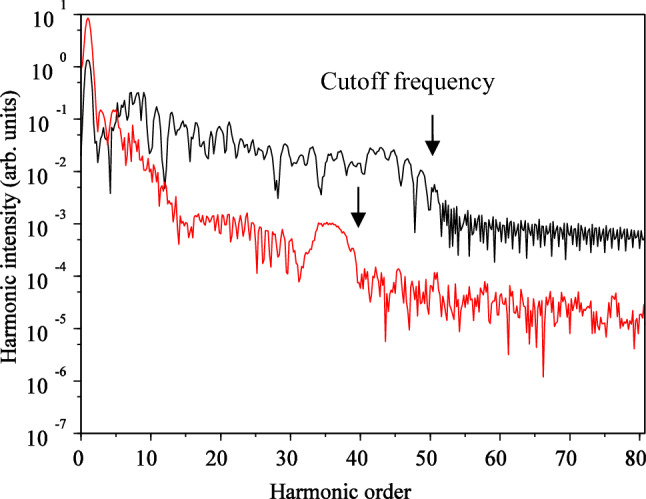
High-order harmonic spectrum on the logarithmic scale for linear (black) and circular (red) polarizations without considering the magnetic field**.** The electric field direction is along the ${\mathbf{x}}$ axis for linear polarization and in the ${\mathbf{xy}}$-plane for circular polarization. The ${\mathbf{x}}$ and ${\mathbf{y}}$ intervals are the same as $\left[ { - 75, 75} \right]$, and the wavelength is $800 {\mathbf{nm}}$ with an intensity of $3.5 \times 10^{14} \;\;{\mathbf{w}}/{\mathbf{cm}}^{2}$.

By substituting the wave function calculated from Eq. (1) into Eq. (10) and then taking the Fourier transform, we can determine the HHG spectrum. The results of HHG for two different polarizations of the interacting field are illustrated in Fig. 4. Besides the shape of the spectrum, which should match experimental results, an essential aspect is the cutoff frequency. Upon examining the outcomes, we observe that for linear polarization, the cutoff frequency precisely aligns with the results obtained from the semi-classical three-step model. For an intensity of $3.5 \times 10^{14} \;\;{\text{W}}/{\text{cm}}^{2}$ and a wavelength of ${\uplambda } = 800\;\;{\text{nm}}$, the classical relation yields a value of $51{\upomega }_{0}$, which is consistent with our quantum numerical simulation results.

However, this agreement is not observed for circular polarization, where our calculations show a lower cutoff frequency. This difference in cut off frequency was also observed by Yuan et al.^55^. The three-step model does not provide an answer to explain this discrepancy. Now, the question arises as to why the quantum model yields a lower value, and in what scenario the cutoff frequency of the quantum model for circular polarization equals that of the linear field. We find the answer to this question when we consider the magnetic field in our calculations.

Another significant result is the difference in HHG efficiency between the linear and circular polarization, with linear polarization exhibiting higher efficiency. This observation is consistent with most modeling studies, and it is attributed to the lower probability of electron collision with the parent nucleus in the interaction of the intense laser pulse with the atom in circular polarization.

**Figure 5:**
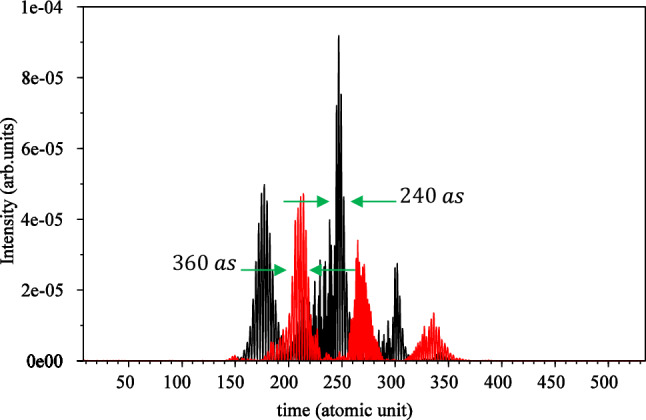
Attosecond pulse train for linear (black) and circular polarization (red) $\times 10^{3}$ without considering the magnetic field. The electric field direction is along the ${\mathbf{x}}$ axis for linear polarization and in the xy-plane for circular polarization. The ${\mathbf{x}}$ and ${\mathbf{y}}$ intervals are both $\left[ { - 75, 75} \right]$, and the wavelength is $800 \;\;{\mathbf{nm}}$ with an intensity of $3.5 \times 10^{14} \;\;{\mathbf{w}}/{\mathbf{cm}}^{2}$.

To calculate the attosecond pulse train, we first define a window for the plateau part of the HHG spectrum, which we choose from $15{\upomega }_{0}$ to $51{\upomega }_{0}$. Then, we use the Fourier series relation given in Eq. (11). The results of these calculations for linear polarization are depicted by a black curve shown in Fig. 5, while the results for circular polarization are represented by a red curve, multiplied by $10^{3}$ for comparison. Each graph contains three peaks.

Apart from the efficiency, the plots clearly demonstrate that the FWHM for the largest peak of the attosecond pulse is smaller for linear polarization compared to circular polarization. Quantitatively, the width of the large attosecond peak is $240{\text{ as}}$ for linear polarization and $360{\text{ as}}$ for circular polarization. This discrepancy is expected, as the circular polarization electric field rotates the electron cloud around the nucleus, leading to different locations for attosecond bursts compared to linear polarization.

According to the picture given by the quantum mechanical model of the interaction of the laser field with the atom, attosecond bursts for circular polarization occur at a greater distance from the mother ion.

In the subsequent steps, we generate the isolated attosecond pulse (IAP) using a method similar to obtaining an APT. The key distinction lies in the frequency window range: for the APT, we consider the plateau region, whereas for the IAP, we focus on the cutoff region.

**Figure 6:**
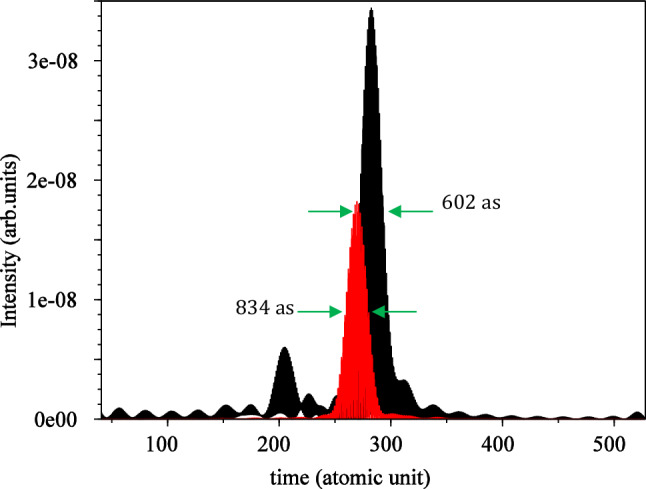
Isolated attosecond pulse for linear (black) and circular polarization (red) without considering the magnetic field. The electric field direction is along the ${\mathbf{x}}$ axis for linear polarization and in the xy-plane for circular polarization. The ${\mathbf{x}}$ and ${\mathbf{y}}$ intervals are both $\left[ { - 75, 75} \right]$, and the wavelength is $800 \;\;{\mathbf{nm}}$ with an intensity of $3.5 \times 10^{14} \;\;{\mathbf{w}}/{\mathbf{cm}}^{2}$.

For linear polarization, we choose a frequency window ranging from $51{\upomega }_{0}$ to $57{{ \omega }}_{0}$, while for circular polarization (red curve), the frequency window is selected from $35{\upomega }_{0}$ to $41{\upomega }_{0}$. Using these frequency windows, we obtained the IAP for the both linear and circular polarizations. As illustrated in Fig. 6, the FWHM for the isolated attosecond pulse is $602\mathrm{ as}$ for linear polarization and $834\mathrm{ as}$ for circular polarization. This comparison clearly indicates the superiority of linear polarization over circular polarization in terms of achieving a narrower attosecond pulse, resulting in a more compact and well-defined temporal structure. The isolated attosecond pulse is a valuable tool for probing ultrafast processes, and the advantage of linear polarization in obtaining a narrower pulse enhances its utility in various applications requiring precise temporal resolution.

### Hydrogen’s wave function, electron trajectory, HHG, APT, and IAP with considering the magnetic field

In the majority of studies concerning the interaction of high-intensity laser pulses with atoms, the magnetic field accompanying the laser pulse is often neglected. This could be due to the increased complexity of calculations or the perception that its effects are minor in the results.

In this section, we examine the impact of the magnetic field on various aspects, including the electron probability density distribution around the nucleus, the HHG, the APT, IAP, and the electron trajectory. We then compare these results with the previous section where the magnetic field is neglected.

To incorporate the magnetic field in the Schrödinger equation, we first calculate the vector potential, ${\vec{\text{A}}}$. Since ${\vec{\text{B}}} = \nabla \times {\vec{\text{A}}}$, and ${\vec{\text{E}}} = - \left( {\partial {\vec{\text{A}}}} \right)/\partial {\text{t}}$, according to Maxwell's equations, for linearly polarized field with ${\vec{\text{E}}}$ in the ${\text{x}}$-direction, ${\vec{\text{A}}}$ lies in the $- {\text{x}}$ direction, resulting in a magnetic field in the $- {\text{y}}$ direction. The electron's quiver motion aligns with the electric field ($+ {\text{x}}$ direction). Consequently, the direction of the electron's quiver velocity (${\vec{\text{v}}}$) is also in the $+ {\text{x}}$ direction. Therefore, the Lorentz force due to the magnetic field, ${\vec{\text{F}}} = {{{\text e} \vec{v}}} \times {\vec{\text{B}}}$, is in the $- {\text{z}}$ direction. Classically, one would expect that the electron would move in the ${\text{xz}}$-plane. Quantum mechanically, the electron trajectory, obtained through the expectation value of the electron's position between quantum states of the electron, also lies in the ${\text{xz}}$-plane. However, for circular polarization, where ${\vec{\text{E}}}$ rotates in the ${\text{xy}}$-plane, the magnetic field associated with the electric field also rotates in the ${\text{xy}}$-plane with a $90^\circ$ phase difference. In this case, the net motion of the electron would be a complex helix, which is a combination of a circular motion which is not closed and is moving on the xy-plane due to electric field, and a helical path due to magnetic field. All boundary conditions are the same as in the previous section, just ${\text{z}}$ interval is ${ }\left[ { - 50,50} \right]{\text{a}}.{\text{u}}.$.

**Figure 7:**
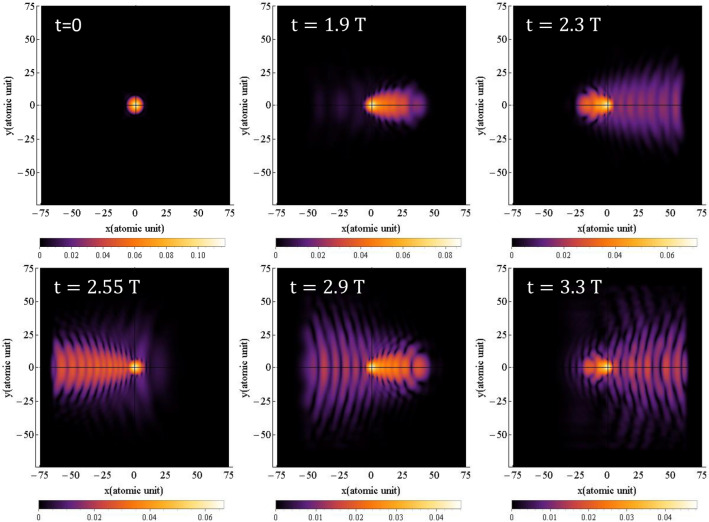
Probability density distribution of electron for linear polarization at various times when magnetic field is considered. The direction of the electric field is along the ${\mathbf{x}}$ axis and direction of the magnetic field is along the ${-}{\mathbf{y}}$ axis. The x and y intervals are both $\left[ { - 75, 75} \right]$ and the ${\mathbf{z}}$ interval is $\left[ { - 50, 50} \right]$. The wavelength is $800 \;\;{\mathbf{nm}}$ with an intensity of $3.5 \times 10^{14} \;\;{\mathbf{w}}/{\mathbf{cm}}^{2}$.

**Figure 8:**
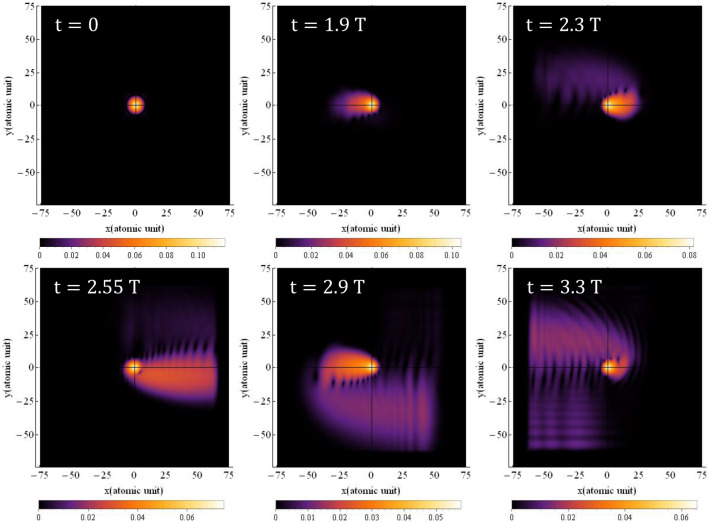
Probability density distribution of electron for circular polarization at various times when magnetic field is considered. The direction of the electric field is in the ${\mathbf{xy}}$-plane and the magnetic field is in the ${\mathbf{xy}}$-plane with a 90° rotation. The ${\mathbf{x}}$ and ${\mathbf{y}}$ intervals are both $\left[ { - 75, 75} \right]$ and the ${\mathbf{z}}$ interval is $\left[ { - 50, 50} \right]$. The wavelength is $800\;\; {\mathbf{nm}}$ with an intensity of $3.5 \times 10^{14} \;\;{\mathbf{w}}/{\mathbf{cm}}^{2}$.

Figures 7 and 8 illustrate the time-dependent probability density distributions for linear and circular polarizations, respectively, when the magnetic field is considered, which are obtained by solving Eq. (1). As shown in Fig. 7, when the magnetic field is considered (see also the supplementary material: video_3 and video_4), the probability density for linear polarization oscillates along a straight line. In comparison to Fig. 1, where the magnetic field is not considered, the results of the probability density distribution are somehow different. Precise speaking, the magnetic term has led to a little bit expansion of the electron probability density in the transverse direction, and so decreases the HHG cut-off frequency and the efficiency. In Fig. 7, the Lorentz force caused by the magnetic field is perpendicular to the plane, although it is not shown in the figure.

**Figure 9:**
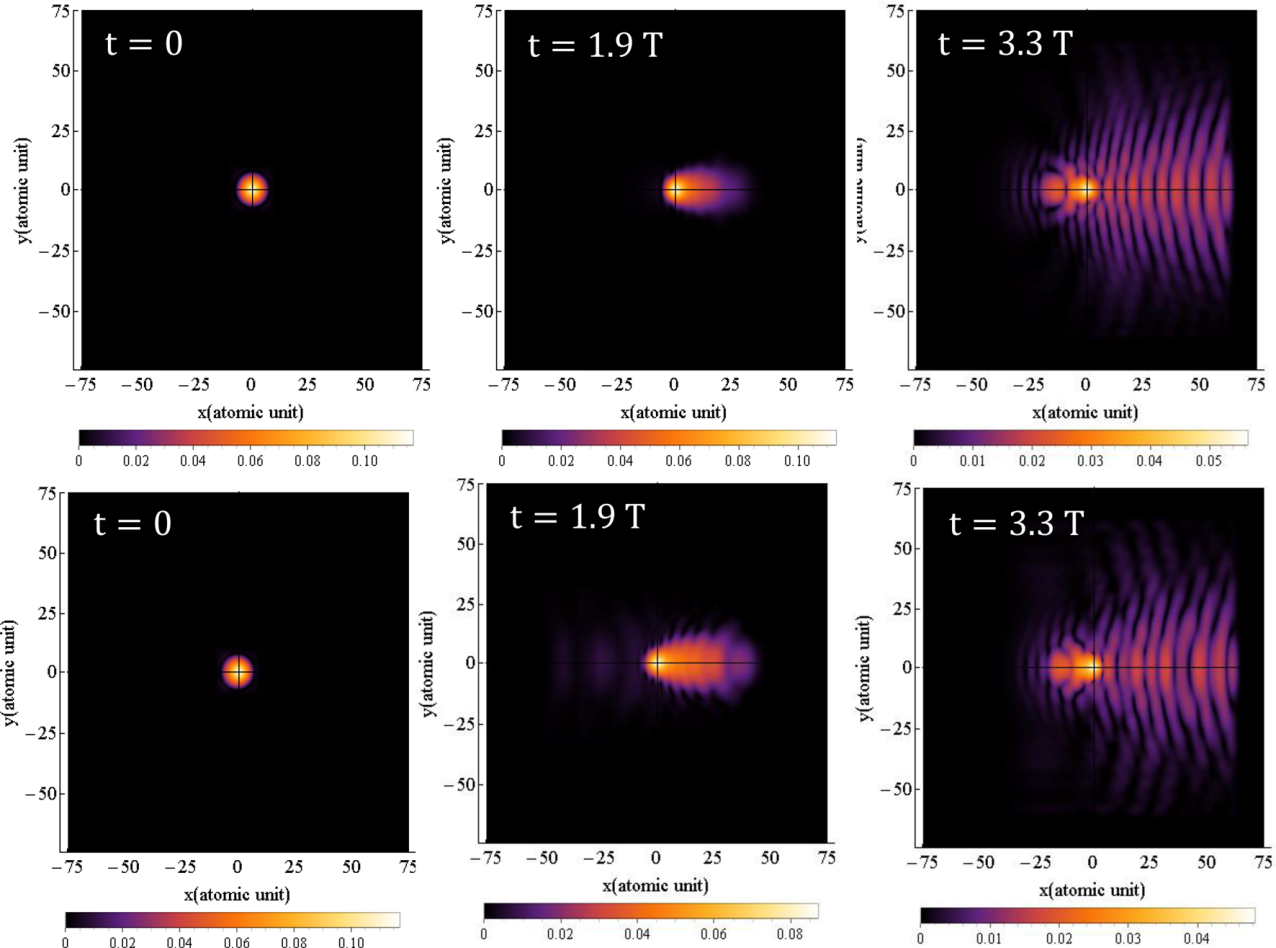
The probability density of the electron around the nucleus with and without considering the magnetic field for linear polarization is given for ${{\varvec{\uplambda}}} = 800 \;\;{\mathbf{nm}}$ and the intensity of $3.5 \times 10^{14} \;\;{\mathbf{W}}/{\mathbf{cm}}^{2}$. The direction of the electric field is along the ${\mathbf{x}}$ axis. The top row is without considering the magnitude field and the bottom row is with the magnetic field.

For a better representation, Fig. 9 displays the electron probability density with and without considering the magnetic field for linear polarization at three different times: $0,{ }1.9{\text{ T}}$ and $3.3{\text{ T}}$. The top row shows the electron probability density without considering the magnetic field, while the bottom row shows the electron probability density with the magnetic field taken into account.

**Figure 10:**
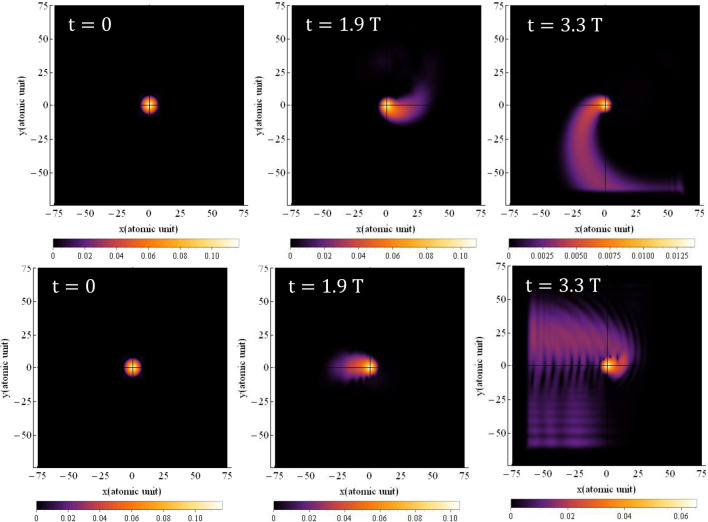
The probability density of the electron around the nucleus with (top) and without (bottom) considering the magnetic field for circular polarization at the wavelength of ${{\varvec{\uplambda}}} = 800 \;\;{\mathbf{nm}}$ and the intensity of $3.5 \times 10^{14} \;\;{\mathbf{W}}/{\mathbf{cm}}^{2}$. The electric field is in xy-plane. The ${\mathbf{x}}$ and ${\mathbf{y}}$ intervals are the same as $\left[ { - 75, 75} \right]$.

In comparison to the linear case, the difference with the non-magnetic case is more pronounced for circular polarization. This implies that the results shown in Fig. 8 exhibit a greater disparity with the results of Fig. 2 (non-magnetic case). Furthermore, in contrast to the non-magnetic case, the oscillations of the probability density function are more prominent in this scenario. Figure 10 illustrates the electron probability density for circular polarization at three different times: $0,{ }1.9{\text{ T}}$ and $3.3{\text{ T}}$. The top row displays the electron probability density without considering the magnetic field, while the bottom row shows the electron probability density with the magnetic field taken into account.

As shown in Fig. 8, the electron density distribution is more transversely spread, and the electron motion becomes more complex. This complexity arises from the combination of a circular motion in the electric field which is not closed and we can call it “moving circle” and the helical motion due the magnetic field. The helical motion, induced by the magnetic field, adds an additional rotational component to the electron’s trajectory, contributing to the increased complexity and transverse spreading of the probability density distribution.

Overall, the inclusion of the magnetic field in the calculations significantly affects the behavior of the electron probability density and its motion, particularly for circular polarization, leading to more pronounced differences from the non-magnetic cases.

**Figure 11:**
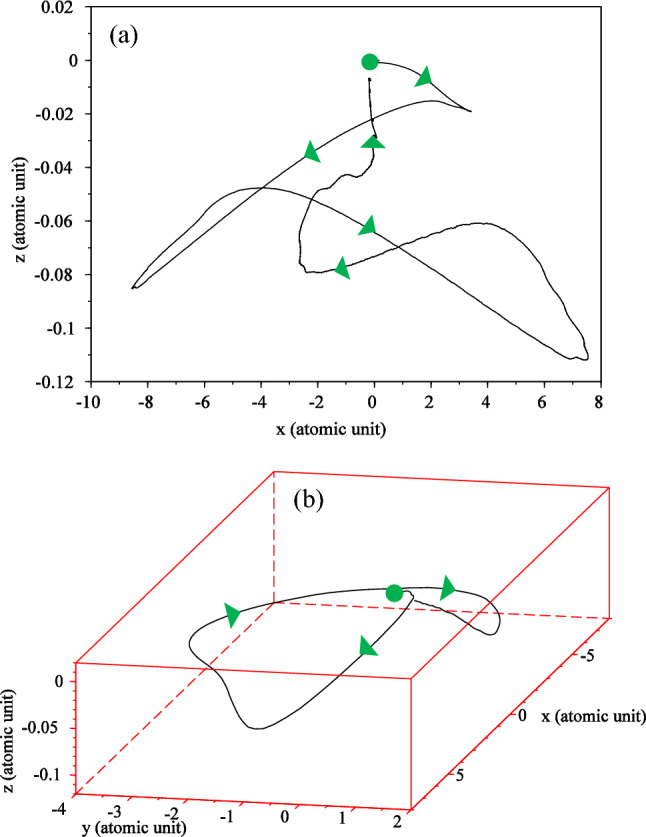
Electron trajectory for (**a**) linear polarization and (**b**) circular polarization for a three-cycle laser pulse with considering the magnetic field. Solid dots depict the starting point, and triangles indicate the direction of electron motion. For linear polarization the electric field direction is along the ${\mathbf{x}}$ axis and direction of the magnetic field is along the ${-}{\mathbf{y}}$ axis. For circular polarization, the electric field is in the ${\mathbf{xy}}$-plane and the magnetic field is in the ${\mathbf{xy}}$-plane with a 90° rotation. The ${\mathbf{x}}$ and ${\mathbf{y}}$ intervals are both $\left[ { - 75, 75} \right]$ and the ${\mathbf{z}}$ interval is $\left[ { - 50, 50} \right]$. The wavelength is $800\;\; {\mathbf{nm}}$ with an intensity of $3.5 \times 10^{14} \;\;{\mathbf{W}}/{\mathbf{cm}}^{2}$.

To provide a more classical and intuitive understanding, we have computed the trajectory of the electron in the presence of a magnetic field for both linear and circular polarizations which is obtained by solving Eq. (8). The results are depicted in Fig. 11.

When a magnetic field is present, a drift motion is added to the electron’s quiver motion. This drift motion causes the electron to move away from the nucleus. In Fig. 11a, we observe the complex path of the electron. Interestingly, even though the polarization is linear, the path of movement becomes intricate due to the existence of the magnetic field. Notably, the electron hits the parent nucleus only once in this case. This behavior demonstrates how the combination of the electric and magnetic fields leads to a unique trajectory for the electron, resulting in a single collision with the parent nucleus. The presence of the magnetic field introduces new dynamics to the electron's motion, adding complexity to its trajectory even for non-relativistic intensities, as seen in our current study.

These findings emphasize the significance of considering the magnetic field in the study of electron dynamics under high-intensity laser pulses, and how it can lead to distinct and intricate motion patterns that impact the overall behavior of the system.

For circular polarization, the results are shown in Fig. 11b. As expected, here the path of the electron is much more complicated than in the linear case. The path of electron movement is shown in three dimensions. Here, a rotational motion caused by the rotating electric field is combined with a meandering motion caused by the rotating magnetic field to form a trajectory that can only be imagined with a diagram. In the motion of the electron around the nucleus, when it has made a circle around the nucleus, near the nucleus, due to the effect of Coulomb attraction, the electron is attracted towards the nucleus and collides with the nucleus. In this movement, the electron hits the parent atom after traveling a round-trip. Our result is in good agreement with the results presented in Ref.^56^, in which authors used strong field approximation.

Although in our quantum model, it is hard to see the fact that the electron hits the nucleus in the presence of a magnetic field, but the calculation of the electron trajectory helps us to easily follow the classical path of the electron according to the three-step model. It is clearly seen that the consideration of the magnetic field complicates the path of the electron, however, the electron hits the mother nucleus after traveling this path. Here we must emphasize that the presence of the magnetic field is not an option, but a requirement, and it is always accompanied by an electric field with a phase difference of 90°.

**Figure 12:**
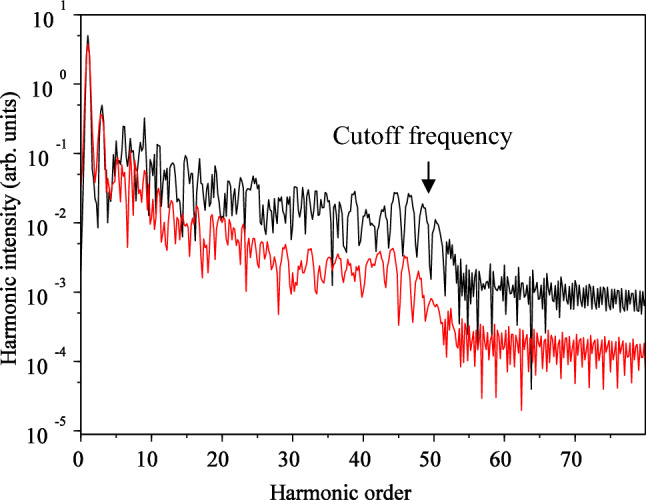
HHG spectrum in the logarithmic scale for the linear (black) and circular (red) polarization, when the magnetic field is considered. For the linear polarization, the electric field direction is along the ${\mathbf{x}}$ axis and direction of the magnetic field is along the ${-}{\mathbf{y}}$ axis. For circular polarization, the electric field is in ${\mathbf{xy}}$-plane, and the magnetic field is also in the ${\mathbf{xy}}$-plane, but with a 90° rotation. The ${\mathbf{x}}$ and ${\mathbf{y}}$ intervals are both $\left[ { - 75, 75} \right]$ and the ${\mathbf{z}}$ interval is $\left[ { - 50, 50} \right]$. The wavelength of the laser pulse is $800 \;\;{\mathbf{nm}}$ with an intensity of $3.5 \times 10^{14} \;\; {\mathbf{W}}/{\mathbf{cm}}^{2}$.

Next, we proceed to calculate the HHG under the influence of the magnetic field, which is obtained by substituting the wave function calculated from Eq. (1) into Eq. (10) and then taking the Fourier transform. The results are presented in Fig. 12. A comparison between Fig. 12 and Fig. 4 reveals that while the appearance of HHG has not changed significantly for linear polarization, there is a significant change for circular polarization. Particularly, the cutoff frequency is shifted for circular polarization. An interesting observation is that the cutoff frequency for circular polarization is close to the cutoff frequency for linear polarization. In other words, in this case, the cutoff frequency for circular polarization follows the cutoff low. It appears that the magnetic field, which complicates the electron's path, may better correct the mismatch of the cutoff frequency results for linear and circular polarizations. This suggests that the three-step model still works well for circular polarization, even though it may not directly explain the electron's collision with the parent atom using the classical picture it creates.

Additionally, in terms of efficiency, the efficiency of both linear and circular polarization cases is close to each other compared to Fig. 4.

We believe and the results show that when the magnetic field is considered, the probability of collision of electron with parent's ion, during this complicated forward and backward motion, increases. The problem would be more understandable when one considers the electron cloud concept, as considered in the quantum mechanical image, rather than the classical paths for the electron. This effect reminds one the “cold cathode vacuum gauge with magnetic field” which measures the vacuum pressure, in which a magnetic field increases the sensitivity of the device, due to increasing the collision of electrons with the anode. In particular, when one considers the problem quantum mechanically, this probability would be more logical and effective.

**Figure 13:**
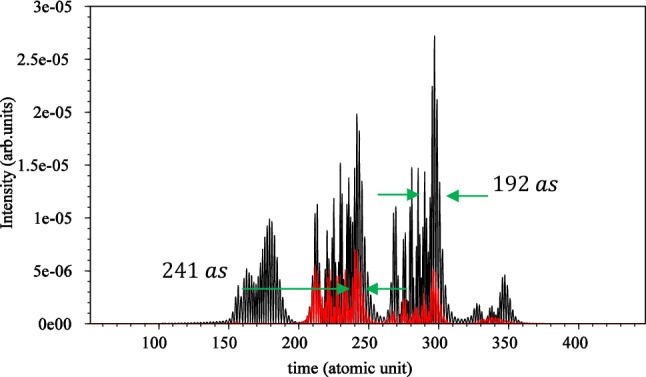
Attosecond pulse train for circular polarization (red) $\times 10$ and linear polarization (black) when the magnetic field is considered. For linear polarization the electric field direction is along the ${\text{x}}$ axis and direction of the magnetic field is along the ${-}{\text{y}}$ axis. For circular polarization the electric field is in the ${\text{xy}}$-plane and the magnetic field is in the ${\text{xy}}$-plane with a 90° rotation. The ${\text{x}}$ and ${\text{y}}$ intervals are both $\left[ { - 75,{ }75} \right]$ and the ${\text{z}}$ interval is $\left[ { - 50,{ }50} \right]$. The wavelength is $800{\text{ nm}}$ with an intensity of $3.5 \times 10^{14} { }\;\;{\text{W}}/{\text{cm}}^{2}$.

By selecting a frequency window from $21{\upomega }_{0}$ to $51{\upomega }_{0}$ and then performing the Fourier transform of the HHG results, we obtain the APT from Eq. (11). Figure 13 illustrates the outcomes of the APT for both linear and circular polarizations. As shown, the FWHM of the attosecond pulse is $192{\text{ as}}$ for linear polarization and $241{\text{ as}}$ for circular polarization. In this figure, the vertical axis is multiplied by $10$ for circular polarization, while in Fig. 4, the vertical axis was multiplied by ${10}^{3}$ for circular polarization.

The results clearly demonstrate that the magnetic field reduces the duration of the main peak of the APT for both polarizations. This effect is observed in both cases. Additionally, the efficiency of the APT generation becomes more comparable for both linear and circular polarizations. These findings further underscore that the consideration of the magnetic field helps bring the results for circular polarization closer to those obtained for linear polarization.

It is important to note that we maintain the principle that the efficiency of APT generation for linear polarization is higher than that for circular polarization. However, the results obtained by considering the magnetic field are not as disparate as the results obtained without accounting for the magnetic field.

**Figure 14:**
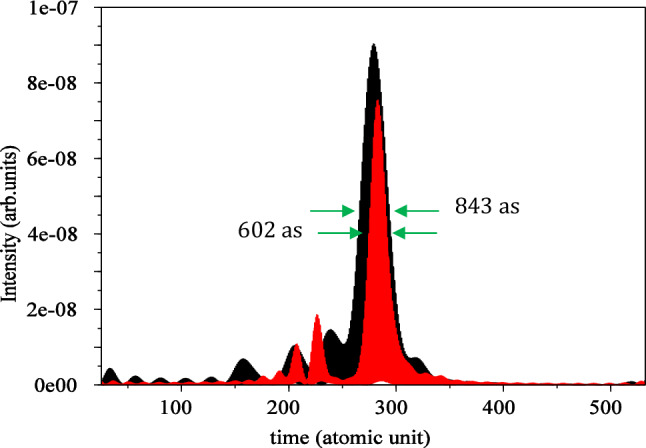
Isolated attosecond pulse for linear (black) and circular polarization (red) $\times 10$ with considering the magnetic field. For linear polarization the electric field direction is along the ${\mathbf{x}}$ axis and direction of the magnetic field is along the ${-}{\mathbf{y}}$ axis. For circular polarization the electric field is in the ${\mathbf{xy}}$-plane and the magnetic field is in the ${\mathbf{xy}}$-plane with a 90° rotation. The x and y intervals are both $\left[ { - 75, 75} \right]$ and the ${\mathbf{z}}$ interval is $\left[ { - 50, 50} \right]$. The wavelength is $800 {\mathbf{nm}}$ with an intensity of $3.5 \times 10^{14} \;\;{\mathbf{W}}/{\mathbf{cm}}^{2}$.

By using a frequency window ranging from $51{\upomega }_{0}$ to $57{{ \upomega }}_{0}$ for linear polarization and $47{\upomega }_{0}$ to $57{\upomega }_{0}$ for circular polarization, we obtain the IAP for both cases from Eq. (11). Figure 14 presents the results, where the FWHM is found to be $843\mathrm{ as}$ for linear polarization and $602\mathrm{ as}$ for circular polarization. The vertical axis is multiplied by $10$ for circular polarization. As the figure shows, when magnetic field is considered, for both cases, clear isolated attosecond pulses cannot be generated. Our attempt to remove the secondary peaks was not successful, and pre-pulses with lower intensities appear beside the main pulse. The shorter pulse duration in circular polarization compared to linear polarization is one of the interesting points of considering the magnetic field.

### Investigating the CEP effect for two cases of considering and not considering the effect of the magnetic field

**Figure 15:**
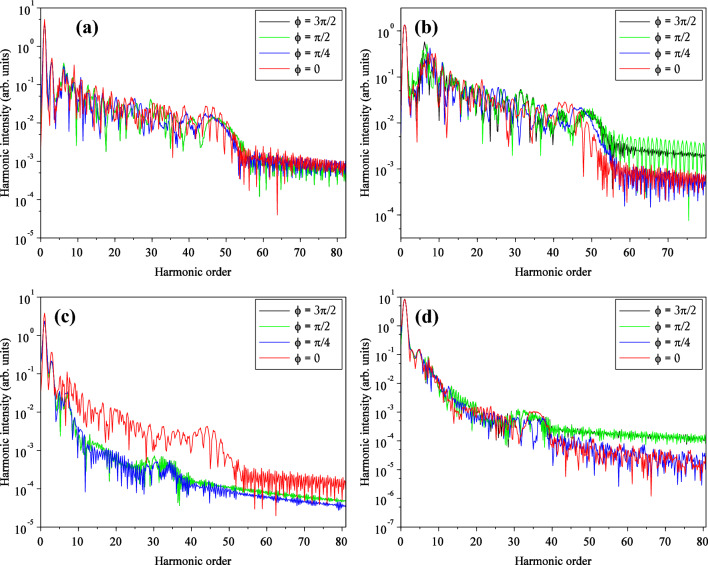
High-order harmonic spectrum in the logarithmic scale for the linear (**a**) and the circular (**c**) polarization, with considering the magnetic field, and for the linear (**b**) and the circular (**d**) polarization without considering the magnetic field. In the case of ignoring magnetic field, the electric field direction is along the ${\mathbf{x}}$ axis for linear polarization and in the ${\mathbf{xy}}$-plane for circular polarization. The ${\mathbf{x}}$ and ${\mathbf{y}}$ intervals are both $\left[ { - 75, 75} \right]$. The wavelength is $800 \;\;{\mathbf{nm}}$ with an intensity of $3.5 \times 10^{14} \;\;{\mathbf{w}}/{\mathbf{cm}}^{2}$ for the laser pulse. In the case of considering magnetic field, for linear polarization the electric field direction is along the ${\mathbf{x}}$ axis and direction of the magnetic field is along the ${-}{\mathbf{y}}$ axis. For circular polarization, the direction of the electric field is in the ${\mathbf{xy}}$-plane and the magnetic field is in the ${\mathbf{xy}}$-plane with a 90° rotation. The other parameters are the same as case without magnetic field.

We have carried out the calculations for three more CEPs; namely $\pi /4$, $\pi /2$, and $3\pi /2$. Figure 15a and c are for linear and circular polarizations, respectively, for the case with magnetic field, and Fig. 15b and d are for linear and circular polarizations, respectively, for the case without magnetic field. For Fig. 15a, the HHG is no longer sensitive to the CEP, but it is the case for the circular polarization. For CEP = $0$, the Fig. 15c shows higher HHG efficiency than other values. Especially, for CEP other than $0$, the HHG shows a smaller cut-off frequency, which is noticeable. For the non-magnetic case, the case of CEP = ${\uppi }/2$ and $3{\uppi }/2$ show higher HHG efficiencies for both, linear and circular polarizations. For linear polarization, Fig. 15b, the cut-off frequency also increases. For circular polarization, as shown in Fig. 15d the cut-off increase is moderate, and its effect is hardly observed.

## Conclusion

In this research, we investigated the interaction of high-intensity femtosecond laser pulses with hydrogen atoms in three dimensions, with and without considering the magnetic field. The time-dependent Schrödinger equation was numerically solved to study the various aspects of the interaction. We explored the electron probability density, high-order harmonics generation, attosecond pulse train, isolated attosecond pulse, and electron trajectory by considering the laser pulses with the wavelength of $800{\text{ nm}}$ and intensity of $3.5 \times 10^{14} {\text{ W}}/{\text{cm}}^{2}$. Although the intensity used was not relativistic, we observed significant corrections in the results, when considering the magnetic field. Considering the magnetic field in the interaction for ultrashort laser pulses, especially for circular polarization, creates very complicated paths for the electron, which is difficult to classically describe as the re-collision of the electron with the atom after changing the direction of the field. But in our quantum model, when the expectation value of the electron's position in the interaction was calculated, it clearly and accurately represented the collision of the electron with the parent nucleus, after traveling a complicated path. In fact, the Coulomb potential which would be important in the vicinity of the nucleus, causes hitting the nucleus by the electron.

When the magnetic field was not considered, the cutoff frequency of high-order harmonics was higher for linear polarization than for circular polarization. In this case, the cutoff frequency for linear polarization was consistent with the cutoff frequency relation obtained from the three-step model, but not for circular polarization. Considering the magnetic field, both cutoff frequencies were almost equal and corresponded to the classical cutoff frequency relation. In general, the production efficiency of high-order harmonics was higher for linear polarization than for circular polarization. However, if the magnified field is not considered, the difference is three orders of magnitude. By including the magnetic field in the calculations, the difference reaches one order of magnitude. In general, the attosecond pulse train width was shorter for linear polarization than for circular polarization. However, when the magnetic field is considered, the attosecond pulse train width is shorter for both polarizations than when the magnetic field is neglected. In the case of the IAP, only the pulse width was reduced for circular polarization; however, in both cases, the production efficiency of the isolated attosecond pulse in linear polarization is higher than that in circular polarization. In this work, the effects of carrier envelope phase (CEP) were also investigated. For circular polarization, when magnetic field presents, CEP = 0 yielded higher efficiency for HHG, but lower cutoff frequency, compared to than other values. For the case of non-magnetic field, CEP= $\uppi /2$ and $3\uppi /2$ showed higher HHG efficiencies and cutoff frequency.

The most significant result of this study is that the quantum mechanical analysis of the interaction between a circularly polarized laser pulse and an atom, considering the magnetic field, provides a better explanation for the results obtained from the classical three-step model. The three-step model is not very convincing in describing the interaction of circularly polarized fields with atoms. The inclusion of the magnetic field in the quantum model offers a more accurate and comprehensive understanding of the electron's behavior and collision dynamics, bridging the gap between quantum and classical descriptions of the interaction process.

## Theory and method of solution

### 3D numerical solution of TDSE

In this section, we present our numerical method used for solving the 3D-TDSE that describes the time evolution of a quantum state. The 3D-TDSE is given by:

1 $${\text{i}}\frac{\partial }{{\partial {\text{t}}}}{\uppsi }\left( {{\vec{\text{r}}},{\text{t}}} \right) = {{{\text H}\psi }}\left( {{\vec{\text{r}}},{\text{t}}} \right),$$

where H is the Hamiltonian of the interacting system. For the case of a hydrogen atom in an external laser field with considering the magnetic field in the Coulomb gauge, the Hamiltonian is given by^57^:

2 $${\text{H}} = \frac{1}{2}\left[ {{\vec{\text{P}}} + {\vec{\text{A}}}\left( {{\vec{\text{r}}},{\text{t}}} \right)} \right]^{2} - {\text{V}}\left( {{\vec{\text{r}}}} \right) = \frac{{{\text{P}}^{2} }}{2} + {\text{A}} \cdot {\text{p}} + \frac{{{\text{A}}^{2} }}{2} - {\text{V}}\left( {{\vec{\text{r}}}} \right),$$

In the above equations, ${\vec{\text{r}}}\left( {{\text{x}},{\text{y}},{\text{z}}} \right)$ and ${\vec{\text{p}}}\left( {{\text{p}}_{{\text{x}}} ,{\text{p}}_{{\text{y}}} ,{\text{p}}_{{\text{z}}} } \right)$ are the position and momentum operators of the electron, respectively. ${\text{P}}^{2} = - \nabla^{2}$, and ${\text{V}}\left( {{\vec{\text{r}}}} \right)$ represents the Coulomb potential, which is smoothed as ${\text{V}}\left( {{\vec{\text{r}}}} \right) = \frac{1}{{\sqrt {{\vec{\text{r}}}^{2} + {\text{a}}^{2} } }}$. The parameter “a” is used to remove the singularity at the origin (${\vec{\text{r}}} = 0$)^16^, with a value of ${\text{a }} = { }0.01$.

${\vec{\text{A}}}\left( {{\vec{\text{r}}},{\text{t}}} \right) = - \smallint {\vec{\text{E}}}\left( {{\vec{\text{r}}},{\text{t}}} \right){\text{dt,}}$ represents the vector potential, where ${\vec{\text{E}}}\left( {{\vec{\text{r}}},{\text{t}}} \right)$ is the external electric field, assumed to be a plane wave propagating along the ${\text{z}}$-direction and given by:

3 $$\vec{E}\left( {z,t} \right) = E_{0} \left[ {C\sin \left( {\omega t + kz + \phi } \right)\hat{i} + D\cos \left( {\omega t + kz + \phi } \right)\hat{j}} \right]f\left( {z,t} \right),$$

Here, $${\text{E}}_{0}$$ is the amplitude of the driving laser field, $$\phi$$ is the carrier envelope phase (CEP), and $$f\left( {{\text{z}},{\text{t}}} \right) = {\text{sin}}^{5} \left( {{\raise0.7ex\hbox{${{\omega t}}$} \!\mathord{\left/ {\vphantom {{{\omega t}} {10}}}\right.\kern-0pt} \!\lower0.7ex\hbox{${10}$}} + {\uppi } + {\text{kz}}} \right)$$ is its envelope function, where $${\text{k}} = {\upomega }/{\text{c}}$$ represents the wave vector with $${\upomega }$$ being the frequency of the driving laser field. The reason for choosing this specific pulse envelope is that although in most cases, the interaction environment is Gaussian, but considering this function in the calculations and requiring that vector potential, $${\vec{\text{A}}}$$, is calculated by the time integration of the driving laser field, we avoid to face with error functions. The $${\text{sin}}^{5} ()$$ function is well-fitted to the Gaussian function, providing an efficient and accurate representation of the laser pulse.

To initiate our numerical calculation, we discretize the time derivative part of the Schrödinger equation as follows:

4 $$\frac{{\partial {\uppsi }\left( {{\vec{\text{r}}},{\text{t}}} \right)}}{{\partial {\text{t}}}} = \frac{{{\uppsi }_{{{\text{m}},{\text{o}},{\text{p}}}}^{{{\text{n}} + 1}} - {\uppsi }_{{{\text{m}},{\text{o}},{\text{p}}}}^{{{\text{n}} - 1}} }}{{2{\Delta t}}}$$

where ${\text{m}},{\text{o}},$, and ${\text{p}}$ are the spatial counters, ${\text{n}}$ is the time counter, and ${\Delta t}$ is the time mesh. For the spatial derivative, we utilize the following discretization:

5 $$\begin{aligned} \nabla^{2} {\uppsi }\left( {{\vec{\text{r}}},{\text{t}}} \right) & = - \left( {\frac{{{\uppsi }_{{{\text{m}} + 1,{\text{o}},{\text{p}}}}^{{\text{n}}} - 2{\uppsi }_{{{\text{m}},{\text{o}},{\text{p}}}}^{{\text{n}}} + {\uppsi }_{{{\text{m}} - 1,{\text{o}},{\text{p}}}}^{{\text{n}}} }}{{{\Delta x}^{2} }}} \right) - \left( {\frac{{{\uppsi }_{{{\text{m}},{\text{o}} + 1,{\text{p}}}}^{{\text{n}}} - 2{\uppsi }_{{{\text{m}},{\text{o}},{\text{p}}}}^{{\text{n}}} + {\uppsi }_{{{\text{m}},{\text{o}} - 1,{\text{p}}}}^{{\text{n}}} }}{{{\Delta y}^{2} }}} \right) \\ & \;\;\;\; - \left( {\frac{{{\uppsi }_{{{\text{m}},{\text{o}},{\text{p}} + 1}}^{{\text{n}}} - 2{\uppsi }_{{{\text{m}},{\text{o}},{\text{p}}}}^{{\text{n}}} + {\uppsi }_{{{\text{m}},{\text{o}},{\text{p}} - 1}}^{{\text{n}}} }}{{{\Delta z}^{2} }}} \right) \\ \end{aligned}$$

where ${\Delta x},{{ \Delta y}}$, ${\Delta z}$ are the spatial meshes. Substituting Eqs. (4) and (5) in Eq. (1), we obtain:

6 $$\begin{aligned} i\frac{{\psi _{{m,o,p}}^{{n + 1}} - \psi _{{m,o,p}}^{{n - 1}} }}{{2\Delta t}} & = - \frac{1}{2}\left( {\frac{{\psi _{{m + 1,o,p}}^{n} - 2\psi _{{m,o,p}}^{n} + \psi _{{m - 1,o,p}}^{n} }}{{\Delta x^{2} }}} \right) - \frac{1}{2}\left( {\frac{{\psi _{{m,o + 1,p}}^{n} - 2\psi _{{m,o,p}}^{n} + \psi _{{m,o - 1,p}}^{n} }}{{\Delta y^{2} }}} \right) \\ & \;\; - \frac{1}{2}\left( {\frac{{\psi _{{m,o,p + 1}}^{n} - 2\psi _{{m,o,p}}^{n} + \psi _{{m,o,p - 1}}^{n} }}{{\Delta z^{2} }}} \right) - \frac{1}{{\sqrt {\left( {x_{0} + m\Delta x} \right)^{2} + \left( {y_{0} + o\Delta y} \right)^{2} + \left( {z_{0} + p\Delta z} \right)^{2} + a^{2} } }}~\psi _{{m,o,p}}^{n} \\ & \;\;\; + iA_{x} \left( {\frac{{\psi _{{m + 1,o,p}}^{n} - \psi _{{m - 1,o,p}}^{n} }}{{2\Delta x}}} \right) + iA_{y} \left( {\frac{{\psi _{{m,o + 1,p}}^{n} - \psi _{{m,o - 1,p}}^{n} }}{{2\Delta y}}} \right) + \frac{{A^{2} }}{2}\psi _{{m,o,p}}^{n} \\ \end{aligned}$$

Simplifying Eq. (6), we have:

7 $$\begin{aligned} \psi _{{m,o,p}}^{{n + 1}} & = - i\left[ {\left\{ { - \frac{1}{2}\left( {\frac{{\psi _{{m + 1,o,p}}^{n} - 2\psi _{{m,o,p}}^{n} + \psi _{{m - 1,o,p}}^{n} }}{{\Delta x^{2} }}} \right) - \frac{1}{2}\left( {\frac{{\psi _{{m,o + 1,p}}^{n} - 2\psi _{{m,o,p}}^{n} + \psi _{{m,o - 1,p}}^{n} }}{{\Delta y^{2} }}} \right)} \right.} \right. \\ & \;\;\; - \frac{1}{2}\left( {\frac{{\psi _{{m,o,p + 1}}^{n} - 2\psi _{{m,o,p}}^{n} + \psi _{{m,o,p - 1}}^{n} }}{{\Delta z^{2} }}} \right) - \frac{1}{{\sqrt {\left( {x_{0} + m\Delta x} \right)^{2} + \left( {y_{0} + o\Delta y} \right)^{2} + \left( {z_{0} + p\Delta z} \right)^{2} + a^{2} } }}~\psi _{{m,o,p}}^{n} \\ & \;\;\; + iA_{x} \left( {\frac{{\psi _{{m + 1,o,p}}^{n} - \psi _{{m - 1,o,p}}^{n} }}{{2\Delta x}}} \right) + iA_{y} \left( {\frac{{\psi _{{m,o + 1,p}}^{n} - \psi _{{m,o - 1,p}}^{n} }}{{2\Delta y}}} \right) + \left. {\left. {\frac{{A^{2} }}{2}\psi _{{m,o,p}}^{n} } \right\} \times 2\Delta t + \psi _{{m,o,p}}^{{n - 1}} } \right] \\ \end{aligned}$$

where $${\text{x}}_{0} ,{\text{y}}_{0} ,$$ and $${\text{z}}_{0}$$ represent the initial spatial conditions. We assumed that the electron is initially in the $$\left| s \right.$$ state. Additionally, a $${\text{cos}}^{1/8}$$ musk function is applied as the boundary condition to prevent the reflection of the wave function from the boundaries, given by $${\text{cos}}^{{{\raise0.7ex\hbox{$1$} \!\mathord{\left/ {\vphantom {1 8}}\right.\kern-0pt} \!\lower0.7ex\hbox{$8$}}}} \left[ {{\vec{\text{r}}} - {\vec{\text{r}}}_{0} /{\vec{\text{R}}} - {\vec{\text{r}}}_{0} } \right]{\raise0.7ex\hbox{${\uppi }$} \!\mathord{\left/ {\vphantom {{\uppi } 2}}\right.\kern-0pt} \!\lower0.7ex\hbox{$2$}}$$^58^, where $${\text{r}}_{0} = 0.8{\text{ R}}$$ is the point from which the mask is applied and $${\vec{\text{R}}} = \sqrt {{\text{x}}_{{\text{f}}}^{2} + {\text{y}}_{{\text{f}}}^{2} + {\text{z}}_{{\text{f}}}^{2} }$$ is the endpoint of the spatial boundary.

### Electron trajectory

In the quantum mechanical approach, the electron trajectory is defined by the expectation value of the electron's position operator. Using the wave function ${\uppsi }\left( {{\vec{\text{r}}},{\text{t}}} \right)$ obtained in part A, the expectation value of the position operator is given by:

8 $${\vec{\text{r}}}\left( t \right) = \left\langle {{\uppsi }\left( {{\vec{\text{r}}},{\text{t}}} \right)\left| {{\vec{\text{r}}}} \right|{\uppsi }\left( {{\vec{\text{r}}},{\text{t}}} \right)} \right\rangle$$

To calculate the expectation value of the electron's position, we perform the following numerical summation:

9 $$\vec{r}^{n} = \mathop \sum \limits_{{m,o,p}} \psi _{{m,o,p}}^{n} {}^{*}\left[ {\hat{i}\left( {x_{0} + m\Delta x} \right) + \hat{j}\left( {y_{0} + o\Delta y} \right) + \hat{k}\left( {z_{0} + p\Delta z} \right)} \right]\psi _{{m,o,p}}^{n}$$

### HHG, APT, and IAP

The HHG, APT, and IAP can be calculated using ${\uppsi }\left( {{\vec{\text{r}}},{\text{t}}} \right)$ obtained in previous section. In the quantum mechanical approach, high-order harmonics are generated by electron oscillations in an external electric field. In other words, the dipole oscillation with time is the radiation source for the new frequencies. The second derivative of dipole moments is more useful, which is obtained by using Ehrenfest’s theorem as^59^:

10 $$\overrightarrow {{{\ddot{\text{r}}}}} \left( {\text{t}} \right) = {\text{i}}\left[ {{\text{H}},\left[ {{\text{H}},{\vec{\text{r}}}} \right]} \right]$$

By taking the Fourier transform of the dipole acceleration, we can obtain the HHG spectrum in the frequency domain. To generate the attosecond pulse train, we apply a frequency window, which involves keeping the plateau range of HHG and removing the rest. Using a Fourier series, we express the attosecond pulse train as follows:

11 $${\text{I}}\left( {\text{t}} \right) = \left| {\mathop \sum \limits_{{\text{q}}} {\text{A}}_{{\text{q}}} {\text{ e}}^{{{{i\omega }}_{{\text{q}}} {\text{t}}}} } \right|^{2}$$

where ${\text{A}}_{{\text{q}}} = \smallint \overrightarrow {{{\ddot{\text{r}}}}} \left( {\text{t}} \right){\text{e}}^{{ - {{iq\omega t}}}} {\text{dt}}$,^59^, and ${\text{q}}$ is harmonic order number. The method for generating the isolated attosecond pulse remains the same, with the difference being that we retain the cutoff region and filter out the rest of the frequencies^10^.

### Ethics approval and consent to participate

All authors agree to the ethics and consent to participate in this article and declare that this submission follows the policies of *Scientific Reports*. Accordingly, the material is the authors' original work, which has not been previously published elsewhere. The paper is not being considered for publication elsewhere. All authors have been personally and actively involved in substantial work leading to the paper and will take public responsibility for its content.

## Supplementary Information

Supplementary Video 1.

Supplementary Video 2.

Supplementary Video 3.

Supplementary Video 4.

## Data Availability

The datasets used and/or analyzed during the current study are available from the corresponding author on reasonable request.
